# Low soil available phosphorus level reduces cotton fiber length via osmoregulation

**DOI:** 10.3389/fpls.2023.1254103

**Published:** 2023-08-18

**Authors:** Miao Sun, Cangsong Zheng, Weina Feng, Jingjing Shao, Chaoyou Pang, Pengcheng Li, Helin Dong

**Affiliations:** ^1^ State Key Laboratory of Cotton Bio-breeding and Integrated Utilization, Institute of Cotton Research, Chinese Academy of Agricultural Sciences, Anyang, China; ^2^ Western Agricultural Research Center, Chinese Academy of Agricultural Sciences, Changji, China

**Keywords:** cotton, soil available phosphorus, low-phosphorus tolerant ability, osmoregulation, fiber length

## Abstract

**Introduction:**

Phosphorus (P) deficiency hinders cotton (*Gossypium hirustum* L.) growth and development, seriously affecting lint yield and fiber quality. However, it is still unclear how P fertilizer affects fiber length.

**Methods:**

Therefore, a two-year (2019-2020) pool-culture experiment was conducted using the split-plot design, with two cotton cultivars (CCRI-79; low-P tolerant and SCRC-28; low-P sensitive) as the main plot. Three soil available phosphorus (AP) contents (P_0_: 3 ± 0.5, P_1_: 6 ± 0.5, and P_2_ (control) with 15 ± 0.5 mg kg^−1^) were applied to the plots, as the subplot, to investigate the impact of soil AP content on cotton fiber elongation and length.

**Results:**

Low soil AP (P_0_ and P_1_) decreased the contents of the osmotically active solutes in the cotton fibers, including potassium ions (K^+^), malate, soluble sugar, and sucrose, by 2.2–10.2%, 14.4–47.3%, 8.7–24.5%, and 10.1–23.4%, respectively, inhibiting the vacuoles from facilitating fiber elongation through osmoregulation. Moreover, soil AP deficiency also reduced the activities of enzymes participated in fiber elongation (plasma membrane H^+^-ATPase (PM-H^+^-ATPase), vacuole membrane H^+^-ATPase (V-H^+^-ATPase), vacuole membrane H^+^-translocating inorganic pyrophosphatase (V-H^+^-PPase), and phosphoenolpyruvate carboxylase (PEPC)). The PM-H^+^-ATPase, V-H^+^-ATPase, V-H^+^-PPase, and PEPC were reduced by 8.4–33.0%, 7.0–33.8%, 14.1–38.4%, and 16.9–40.2%, respectively, inhibiting the transmembrane transport of the osmotically active solutes and acidified conditions for fiber cell wall, thus limiting the fiber elongation. Similarly, soil AP deficiency reduced the fiber length by 0.6–3.0 mm, mainly due to the 3.8–16.3% reduction of the maximum velocity of fiber elongation (V_Lmax_). Additionally, the upper fruiting branch positions (FB_10–11_) had higher V_Lmax_ and longer fiber lengths under low soil AP.

**Discussion:**

Cotton fibers with higher malate content and V-H^+^-ATPase and V-H^+^-PPase activities yielded longer fibers. And the malate and soluble sugar contents and V-H^+^-ATPase and PEPC activities in the SCRC-28's fiber were more sensitive to soil AP deficiency in contrast to those of CCRI-79, possibly explaining the SCRC-28 fiber length sensitivity to low soil AP.

## 1 Introduction

Phosphorus (P) is one of the three nutrient elements essential for cotton (*Gossypium hirustum* L.) growth and development (Sun et al., 2022). It can stimulate budding and flowering in the middle growth stage and promote the maturity and weight increase of cotton bolls in the late growth stage, thus directly affecting the lint yield and fiber quality (Sun et al., 2018). According to statistics, there are 5.7×10^9^ ha of land with AP deficiency on earth, which affects agricultural production (Cordell et al., 2009; Xu et al., 2020), and about 30% of China’s farmland has only 3−5 mg kg^-1^ of AP (Gao et al., 2019). Cotton production is concentrated in Xinjiang and the saline-alkali areas in China. The soil types of these cotton growing areas are mostly calcareous, with a high P fixation capacity. Coupled with drought and limited rain, the lack of available phosphorus (AP) in the soil is a major problem in these cotton-growing areas (Wang et al., 2010). Therefore, applying a large quantity of P fertilizer is necessary to ensure a high yield and quality of cotton and reduce P fertilizer utilization efficiency in cotton fields (Chen et al., 2020). However, there is a shortage of high-grade P rock resources and a low mining recovery rate of P in China, which may cause a P fertilizer shortage in the future (Zhou et al., 2021).

Cotton is the major industrial crop for natural fiber production (Yang et al., 2023), and cotton textiles are produced primarily in China, which is the world’s largest producer of cotton. The cotton industry provides economic income for cotton farmers and raw materials for the textile industry, thus playing a vital role in the national economy (Yu, 2018). There is increasing demand for high cotton fiber quality. Thus, the cotton plants need sufficient P supply during the whole growth period to ensure lint yield and fiber quality (Chen et al., 2020).

Compared with the cotton plants supplied with 40.3 kg·P_2_O_5_ ha^-1^, the cotton fiber length, strength, and micronaire value of the plants without P treatment decreased by 1.6%, 1.0%, and 2.6%, respectively (Sarkar and Majumdar, 2002). P deficiency (no P) reduced the length (Gutstein, 1970) and strength (Li et al., 2020) of cotton fibers but increased the micronaire value (Li et al., 2020) compared to applying superphosphate or triple superphosphate. However, the P application rate of 0−90 kg·P_2_O_5_ ha^-1^ did not affect the cotton fiber quality indicators (length, strength, and micronaire value) (Mukundan et al., 1990; Tewolde and Fernandez, 2003). In summary, the effects of P fertilizers on cotton fiber quality are inconsistent, probably due to the different cotton varieties (Mukundan et al., 1990; Tewolde and Fernandez, 2003) or the amount/type of P fertilizer used (Gutstein, 1970; Mukundan et al., 1990; Li et al., 2020). These differences might also be related to the soil AP content of the experimental sites.

The leaves subtending cotton bolls provide 60% to 87% of carbon for the cotton bolls, seriously affecting their growth and development (Ashley, 1972; Constable and Rawson, 1980; Wullschleger and Oosterhuis, 1990). Thus, the leaves and bolls are the primary “sources” and “sinks” of photosynthetic products. Cotton yield and quality are influenced by the “subtending leaves - cotton bolls” association (Liu et al., 2014). Our earlier research found that by decreasing sucrose synthesis and transportation in the leaves subtending cotton bolls, there is a decrease in cotton boll biomass and lint yield for two cotton cultivars (CCRI-79; low-P tolerant and SCRC-28; low-P sensitive) when the soil AP content is low (Sun et al., 2022). However, the regulatory mechanisms of fiber quality remain poorly understood.

Cotton fiber cells begin transitioning from expansion elongation (non-polar) to polar elongation at two days post-anthesis (DPA). The fiber cell elongation determines the length of cotton fibers, an important parameter in the textile industry (Yang et al., 2023). Vacuolar turgor supports the elongated fiber cells, and the elongation direction is determined by both turgor pressure and cell wall structure (Mao, 2019). There are four predominant osmotically active solutes in fiber cells: potassium ion (K^+^), malate, soluble sugar, and sucrose. After entering the vacuoles through the reverse content gradient, these solutes exert osmoregulation, causing water to enter the vacuoles and fiber cells during elongation (Ruan et al., 1997; Ruan et al., 2001). The plasma membrane (PM) H^+^-ATPase (PM-H^+^-ATPase) pumps out H^+^, forming transmembrane ion gradients to provide the initial power for the transmembrane transport of osmotically active solutes. Meanwhile, the acidification of the surrounding environment facilitates cell wall expansion. Vacuolar membrane H^+^-ATPase (V-H^+^-ATPase) and H^+^-translocating inorganic pyrophosphatase (H^+^-PPase) play similar roles (Smart et al., 1998). Low P significantly increased sucrose content in cotton leaves (Liu et al., 2021) and the H^+^-ATPase activity of rice roots (Zhang, 2011). Phosphoenolpyruvate carboxylase (PEPC) is the rate-limiting enzyme for malate synthesis in fiber cells (Smart et al., 1998); however, its transcript levels greatly vary under low P conditions. The *PEPC* gene was up-regulated in tobacco (Toyota et al., 2003) but down-regulated in *Arabidopsis* (Wu et al., 2003) under low P conditions. Thus, there is a need to further investigate whether low P would affect the osmotically active solutes’ contents (K^+^, malate, soluble sugar, and sucrose) and the related enzymes’ activities (PM-H^+^-ATPase, V-H^+^-ATPase, V-H^+^-PPase, and PEPC) of cotton fiber cells through the “subtending leaves (source) - cotton fiber (sink)” association. Determining whether these effects impact the cotton fiber length and the mechanisms involved is also important.

Therefore, this research explored (1) elongation and length of cotton fibers in the presence of soil AP deficiency; (2) the relationship between osmotically active solute contents and linked enzymes activities during fiber elongation and leaf P content of subtending leaves; (3) the key osmotically active solutes and enzymes when soil AP is low. Our findings provide a reference for further research on improving fiber qualities in low AP soil.

## 2 Materials and methods

### 2.1 Description of the experimental site

In Anyang (36°06′ N and 114°21′ E), Henan, China, Chinese Academy of Agricultural Sciences’ Institute of Cotton Research carried out a two-year pool-culture study from 2019 to 2020. Each pool was 3.6-m-long, 4-m-wide and 1.5-m-high. The experimental soil type is classified as Inceptisols (USDA Soil Taxonomy). Clay loam was the soil used in the experiment (Li et al., 2017). Among soil layers within 0−20 cm, the organic matter, total nitrogen, and available nitrogen, phosphorus, and potassium were respectively 12.9 g kg^−1^, 0.86 mg kg^−1^, 64.4 mg kg^−1^, 3.1 mg kg^−1^, and 163.6 mg kg^−1^ in 2019 and 13.1 g kg^−1^, 0.85 mg kg^−1^, 63.3 mg kg^−1^, 3.0 mg kg^−1^, and 180.4 mg kg^−1^ in 2020. Meteorological data of the cotton growing seasons in 2019 and 2020 are presented in **Table 1**.

**Table 1:** Anyang experimental station weather data for the growing seasons of 2019 and 2020.

Month	Sunshine duration (h)		Average temperature (°C)		Precipitation (mm)	
	2019	2020	2019	2020	2019	2020
April	193.6	284.4	14.4	14.2	70.4	28.8
May	297.7	294.3	22.0	22.2	5.0	39.5
June	256.7	206.5	27.9	26.2	55.4	50.4
July	260.2	204.4	28.5	26.0	42.0	29.2
August	186.6	196.4	25.5	26.0	116.1	156.1
September	213.7	226.3	22.0	21.9	51.3	3.6
October	139.9	123.1	15.4	14.4	51.5	9.9
Average/total	1548.4	1535.4	22.2	21.6	391.7	317.5

During the experiments, all weather data was collected from an automatic weather station 4 kilometers away.

### 2.2 Management of experimental fields and design of experiments

The research used the split-plot design, with the main plot consisting of the low-P-tolerant cotton cultivar CCRI-79 and the low-P-sensitive cultivar SCRC-28, selected in the previous study (Sun et al., 2022). The subplots contained three soil AP levels; 3 ± 0.5 mg kg^-1^ (P_0_, extreme soil AP deficiency), 6 ± 0.5 mg kg^-1^ (P_1_, moderate soil AP deficiency), and 15 ± 0.5 mg kg^-1^ (P_2_, control). The soils from 20–40 cm depth in the field were chosen to develop P-deficiency in pool soil. Soil AP levels were regulated using triple superphosphate (44% P_2_O_5_) (Sun et al., 2022). The P fertilizer amount of 0, 50.6, and 202.4 g pool^−1^ was applied in P_0_, P_1_, and P_2_ during both years. The nitrogen fertilizer used was 225 kg N ha^-1^ (urea, 46% N), and 50% of the fertilizer was used for basal application before sowing (April 23, 2019, and April 15, 2020), while the other 50% for topdressing in the early flowering stage (July 22, 2019, and July 6, 2020). Furthermore, the potassium fertilizer used was 150 kg K_2_O ha^-1^ (potassium sulfate, 51% K_2_O) and was applied as basal fertilizer (Li et al., 2017). We applied the base fertilizer 7 days before sowing, and we watered the soil to dissolve it. An analysis of soil samples at the 0−20 cm soil layer was conducted one month after the application of base fertilizer at the surface level of the soil, after which the samples were dried and sieved at a fineness of 1 mm. The soil AP contents were determined by the Olsen-P method (Yang and Jacobsen, 1990). Thereafter, cotton seeds were planted through manual drilling (April 30, 2019, and April 24, 2020). Five rows of 80 cm spacing were used in each experimental plot with an area of 14.4 m^2^ and a density of 52,500 plants per hectare. Every treatment was conducted in three replicates, and field management was in accordance with the management measures for high-yielding cotton cultivation.

### 2.3 Sampling and handling

During the flowering period of cotton plants, 8−10 cotton bolls of the first fruit node in the lower fruiting branches (FB_2-3_), middle fruiting branches (FB_6-7_), and upper fruiting branches (FB_10-11_) were collected at 5, 10, 15, 17, 24, 31, 38, and 45 DPA (8:00-9:00 a.m.). The harvested cotton bolls were placed on ice, and the cotton fibers were peeled off from the cotton seeds within one hour. Thereafter, we analyzed the malate content and enzyme activity of one third (1/3) of the cotton fibers that were frozen in liquid nitrogen and held at -80°C. Another 1/3 was dried at 70°C to a constant weight to determine K^+^ and carbohydrate contents. The remaining 1/3 was used for fiber length measurement.

### 2.4 Measuring K^+^, malate, and carbohydrate contents of the cotton fibers

After crushing with a disintegrator, dried cotton fiber samples were sieved at 0.5 mm. Thereafter, K^+^ was extracted from the samples using the H_2_SO_4_-H_2_O_2_ digestion method, and its content was determined by an atomic absorption spectrometer (NOVAA400P, Analytik Jena GmbH, Jena, Germany) at 769.9 nm.

After snap-freezing in liquid nitrogen, 50 mg of frozen fiber samples were ground into powder, and a 1-hour extraction at 80°C was performed on the powder using 1.5 ml of the buffer containing 1.2 ml of absolute ethanol, 100 mM Hepes-KOH (pH 7.1), and 20 mM MgCl_2_ to obtain the crude extract. We centrifuged the crude extract at 12000×g (5 min), and the supernatant was collected and mixed with 150 μl of active carbon (100 mg ml^-1^). The mixture was centrifuged under the same conditions, and the supernatant was collected. Finally, 5 ml of the supernatant was transferred into a 10 ml cuvette, and 1 ml of Tris-HCl (0.2 mol L^-1^ Tris, 0.2 mol L^-1^ HCl, and pH 5.36) was added and mixed. Incubate for 15 min with distilled water topped up to 25 ml. The malate contents were measured on the spectrophotometer (SPECORD 40, Analytik Jena GmbH, Jena, Germany) at 656 nm using the colorimetric method (Zhang et al., 2017).

Weighed (0.1 g) cotton fibers were placed in a 10 ml centrifuge tube with 5 ml of 80% ethanol (v/v) and incubated at 80°C for carbohydrates extraction (30 min). The extracts were centrifuged (10000×g, 5 min), and a 25 ml volumetric flask was used to collect the supernatant. Repetition of the extraction procedure was performed, and the obtained supernatant was topped up to 25 ml with ethanol (80%, v/v). In accordance with Hendrix et al. (1993), we measured the soluble sugar and sucrose contents.

### 2.5 PEPC, PM-H^+^-ATPase, V-H^+^-ATPase, and V-H^+^-PPase activities in cotton fibers

Hu et al. (2018) method was used to measure PEPC, V-H^+^-ATPase, and V-H^+^-PPase activities. Briefly, the fiber powder was extracted with 5 ml of the buffer containing 30 mM Hepes-Tris (pH 7.4), 250 mM mannitol, 3 mM ethylenediaminetetraacetic acid (EDTA), 1 mM phenylmethylsulfonyl fluoride (PMSF), 1.5% (w/v) polyvinylpyrrolidone (PVP) 4000, 1 mM dithiothreitol (DTT), and 0.1% (w/v) bovine serum albumin (BSA). In a centrifuge operated at 4°C, the homogenate was centrifuged (480×g, 10 min) and the supernatants were stored to measure the enzyme activities. The reaction solution (1100 µl) for PEPC activity assay contained 800 μl of the reaction buffer (30 mM Hepes-Tris (pH 7.5), 10 mM MgCl_2_, 10 mM NaHCO_3_, and 0.5 mM DTT), 100 μl of crude enzyme solution, 100 µl of malate dehydrogenase (EC 1.1.1.37, 100 U ml^-1^, SIGMA), and 100 µl of 30 mM PEP. The PEPC activity was measured using the colorimetric method at 340 nm. For V-H^+^-ATPase activity determination, the reaction solution (500 µL) contained 400 μl of the reaction buffer (30 mM Hepes-Tris (pH 7.5), 3 mM MgSO_4_, 50 mM KCl, 0.5 mM NaN_3_, 0.125 mM (NH_4_)_2_ MoO_4_, and 0.125 mM Na_3_VO_4_), 50 μl of crude enzyme solution, and 50 μl of 20 mM ATP-Tris. Half-hour was spent incubating the mixed solution at 30°C. In order to terminate the reaction, 1 ml of the stop solution (5% (NH_4_)_2_MoO_4_: 5 M H_2_SO_4_: H_2_O=1: 1: 3) was added. Finally, 200 μl of chromogenic agent (0.25 g of aminophenol sulfonic acid in 100 ml of 1.5% Na_2_SO_3_ (pH 5.5) mixed with 0.5 g of Na_2_SO_3_) was added and the mixture was incubated for 20 min at 37°C. The V-H^+^-PPase activity was also determined using the colorimetric method at 660 nm. Its reaction solution (500 µl) contained 400 μl of the reaction buffer (30 mM Hepes-Tris (pH 7.5), 3 mM MgSO_4_, 50 mM KCl, 0.5 mM NaN_3_, and 0.125 mM (NH_4_)_2_ MoO_4_), 50 μl of crude enzyme solution, and 50 μl of 20 mM PP-Tris. The following measurement steps were the same as those employed to determine V-H^+^-ATPase activity. PM-H^+^-ATPase was isolated using Hu et al.’s method (2018). In brief, frozen fiber tissues were ground in an ice bath with 5 ml of cold buffer comprising 50 mM Hepes-Tris (pH 7.0), 300 mM sucrose, 8 mM EDTA, 2 mM PMSF, 1.5% (w/v) PVP 4000, 4 mM DTT, and 0.2% (w/v) BSA. In 4°C, the homogenate was centrifuged (10000×g, 20 min). In order to analyze enzyme activity in the supernatants, the supernatants were stored. The reaction solution (500 µL) for PEPC activity assay contained 400 μl of reaction buffer (30 mM Hepes-Tris (pH 6.5), 3 mM MgSO_4_, 50 mM KCl, 0.5 mM NaN_3_, 0.125 mM (NH_4_)_2_ MoO_4_), 50 μl of crude enzyme solution, and 50 µl of 20 mM ATP-Tris. The subsequent measurement processes were in accordance with those of V-H^+^-ATPase activity determination.

### 2.6 Cotton fiber length

Boiling cotton bolls formed before 30 DPA in 0.1% (v/v) HCl separated fibers from cotton seeds (Schubert et al., 1973). The fibers were stretched through the flowing water method (Thaker et al., 1989), and their lengths were measured using a vernier caliper.

For cotton bolls formed after 30 DPA, the fibers were peeled and incubated at 60°C (30 min) and then at 40°C (2 h). The fibers were finally incubated in a standard assay chamber for 48 h, with temperature and humidity set at 20 ± 2°C and 65 ± 5%, respectively. The fiber length was then determined using a photoelectric stapler (Y-146, Taicang Electron Apparatus Co., Ltd., China) (Yang et al., 2016).

The fibers were obtained by ginning seed cotton from cotton bolls harvested on September 15, 2019, and September 15, 2020. After drying the cotton fiber samples to a constant weight at 35°C, the fiber length was analyzed at the Supervision, Inspection, and Test Center of Cotton Quality, Ministry of Agriculture and Rural Affairs, China.

### 2.7 Statistical analysis

In order to calculate means, standard errors, and coefficients of variation (CV, %), Microsoft Excel 2007 (Microsoft Corp., Redmond, WA, USA) was used. The SPSS statistical software Version 23.0 (IBM Corp., New York, NY, USA) was used to conduct a variance analysis for all treatments at a 5% significance level, applying the least significant difference (LSD). In order to analyze each variable’s specific relevance, Pearson correlation coefficient was used.

## 3 Results

### 3.1 Osmotically active solute contents of the fibers

**Figure 1:**
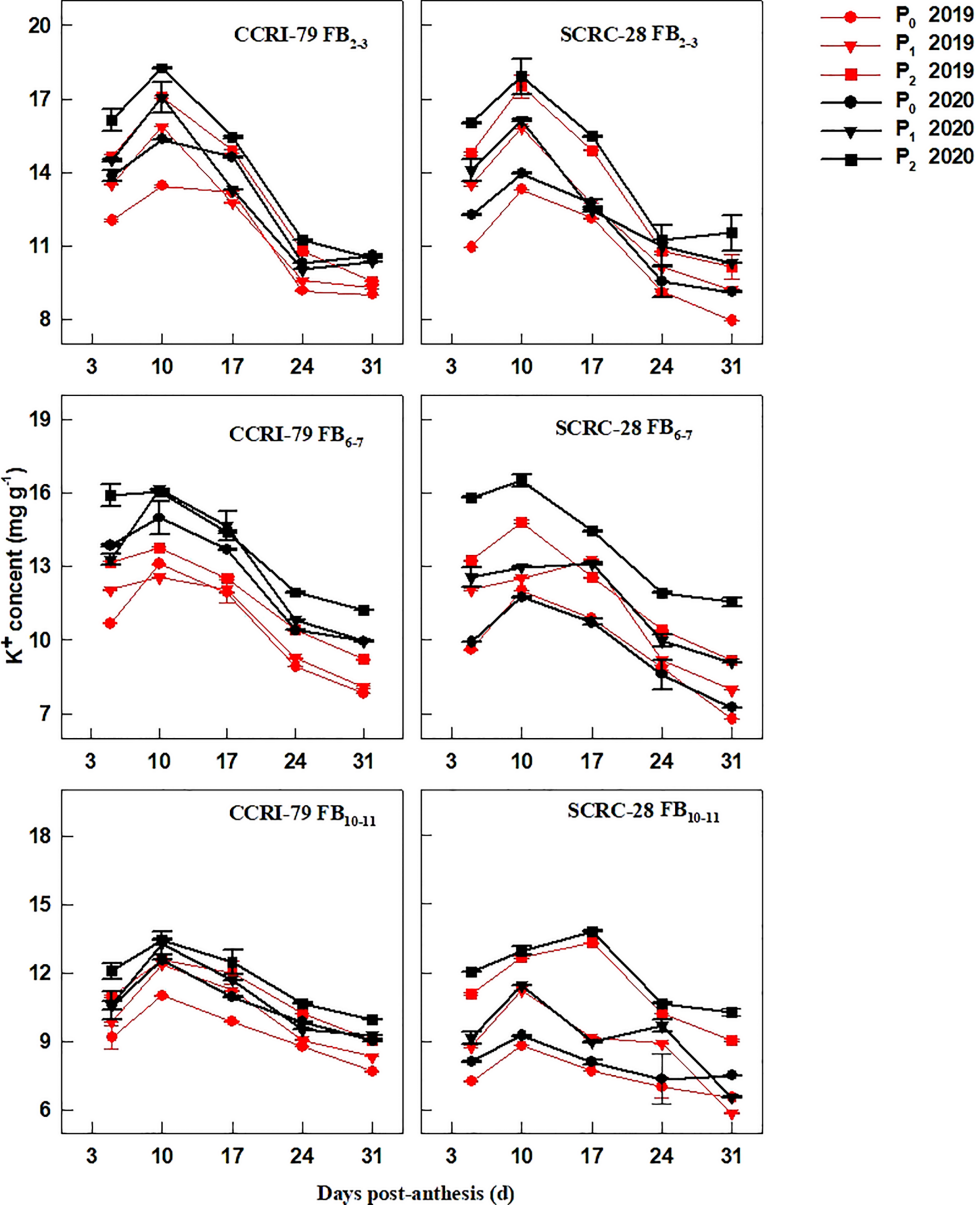
Cotton fiber potassium ion (K^+^) content in relation to soil available phosphorus (AP) levels in 2019 and 2020. FB: fruiting branch. P_0_: 3 ± 0.5 mg kg^−1^. P_1_: 6 ± 0.5 mg kg^−1^. P_2_: 15 ± 0.5 mg kg^−1^. The cotton fibers were collected at 5, 10, 17, 24, and 31 days post-anthesis (DPA) (8:00-9:00 a.m.). Error bars indicate SE (n = 3).

Low soil AP levels (P_0_ and P_1_) decreased the fiber K^+^ contents (**Figure 1**). Under P_1_ and P_0_, the average fiber K^+^ contents reduced by 7.3−8.8% and 10.3−12.3% in CCRI-79 and 10.6−22.7% and 20.8−33.1% in SCRC-28, respectively, at the 3 fruiting branch positions (FBPs) compared to P_2_. Low soil AP had less effect at the FB_10–11_ compared to FB_2–3_ and FB_6–7_ (**Figure 1**). For all soil AP levels, CCRI-79 and SCRC-28 had average fiber K^+^ contents of 11.2-12.7 mg g^-1^ and 9.5-12.9 mg g^-1^, respectively. However, SCRC-28 had a higher CV (15.3%) than CCRI-79 (6.3%). CCRI-79 had fiber K^+^ contents 6.9 and 18.0% higher than SCRC-28 in P_1_ and P_0_, but 1.5% lower in P_2_ (**Figure 1**).

**Figure 2:**
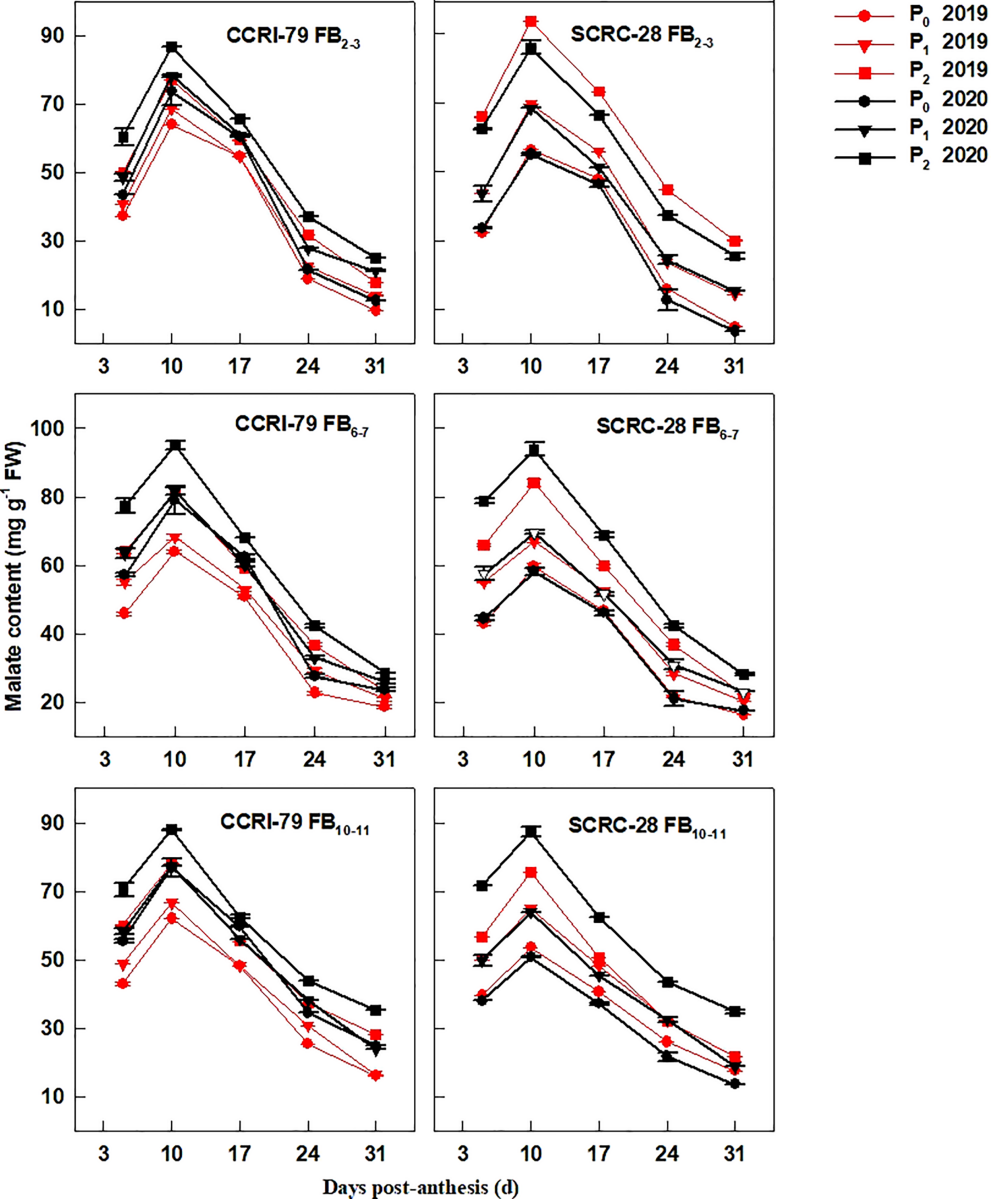
Cotton fiber malate content in relation to soil available phosphorus (AP) levels in 2019 and 2020. FB: fruiting branch. P_0_: 3 ± 0.5 mg kg^−1^. P_1_: 6 ± 0.5 mg kg^−1^. P_2_: 15 ± 0.5 mg kg^−1^. The cotton fibers were collected at 5, 10, 17, 24, and 31 days post-anthesis (DPA) (8:00-9:00 a.m.). Error bars indicate SE (n = 3).

The fiber malate content increased at 5−10 DPA and decreased at 10−31 DPA (**Figure 2**). Under the P_1_ and P_0_ treatments, the malate content decreased by 14.4–17.0% and 20.2–22.5% for CCRI-79, and 20.7–30.0% and 35.4–47.3% for SCRC-28, respectively, across the three FBPs in 2019 and 2020. SCRC-28 had a greater CV of 25.6% compared to CCRI-79’s CV of 6.3%. Additionally, In P_1_ and P_0_, CCRI-79 had malate contents that were 7.9% and 26.5% higher than SCRC-28, respectively, but in P_2_, they were 3.3% lower. At the same soil AP level, fibers from FB_6–7_ and FB_10–11_ had higher malate content in contrast with FB_2–3_ (**Figure 2**). According to the above data, SCRS-28 (low-P sensitive) had greater reductions of K^+^ and malate contents in fibers than CCRI-79 (low-P tolerant) under low soil AP treatments (P_0_ and P_1_).

**Figure 3:**
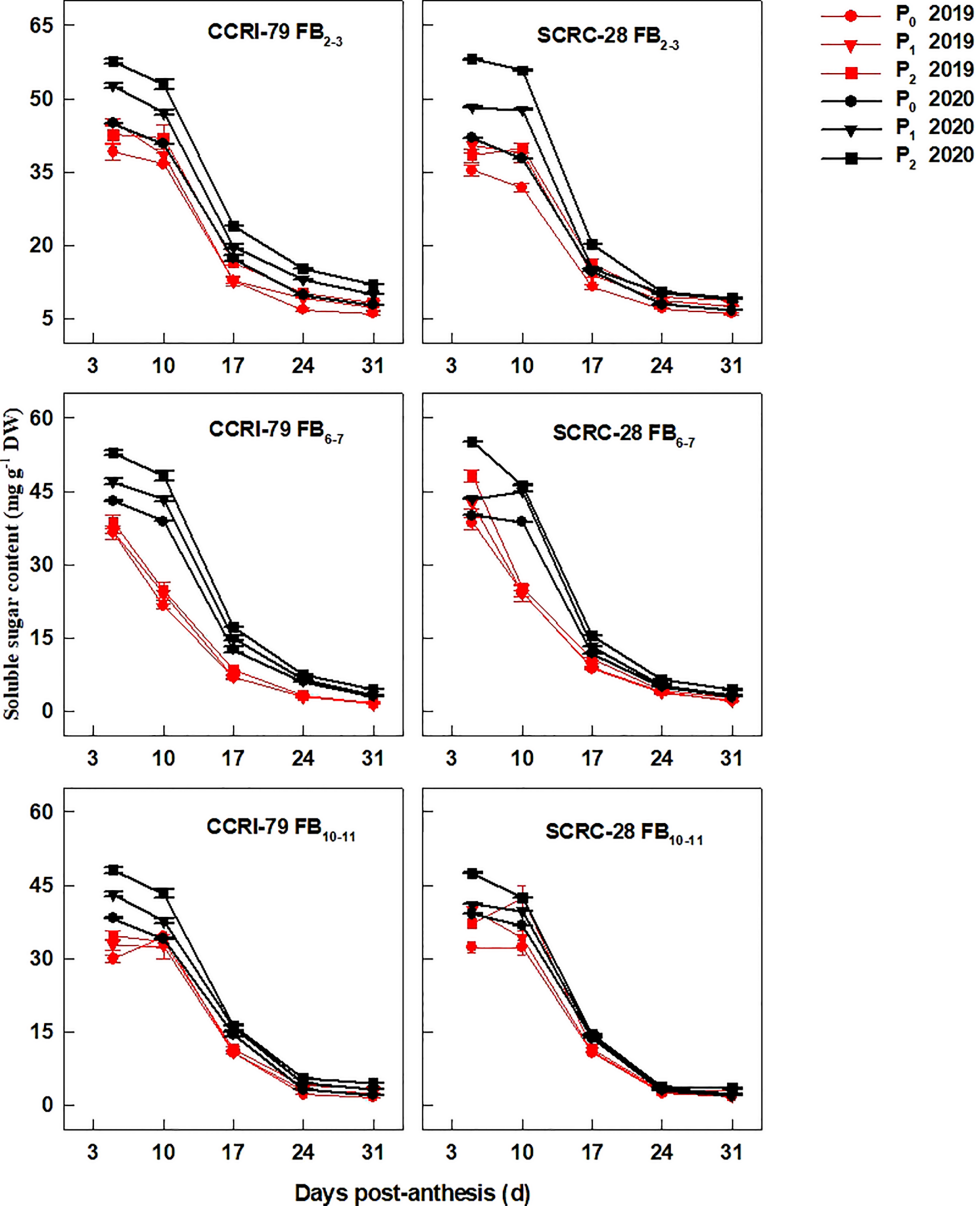
Cotton fiber soluble sugar content in relation to soil available phosphorus (AP) levels in 2019−2020. FB: fruiting branch. P_0_: 3 ± 0.5 mg kg^−1^. P_1_: 6 ± 0.5 mg kg^−1^. P_2_: 15 ± 0.5 mg kg^−1^. The cotton fibers were collected at 5, 10, 17, 24, and 31 days post-anthesis (DPA) (8:00-9:00 a.m.). Error bars indicate SE (n = 3).

Nevertheless, the soluble sugar content of fibers from the three FBPs declined with the development of cotton bolls, especially at 10–31 DPA compared to 5–10 DPA (**Figure 3**). Compared to P_2_, the soluble sugar content reduced by 9.1−9.6% and 16.5−20.9% for CCRI-79, in P_1_ and P_0_ at the three FBPs, respectively, over the two years. A similar change pattern was observed for SCRC-28, with an 8.7−12.4% and 16.5−24.5% decrease under the same conditions (**Figure 3**).

**Figure 4:**
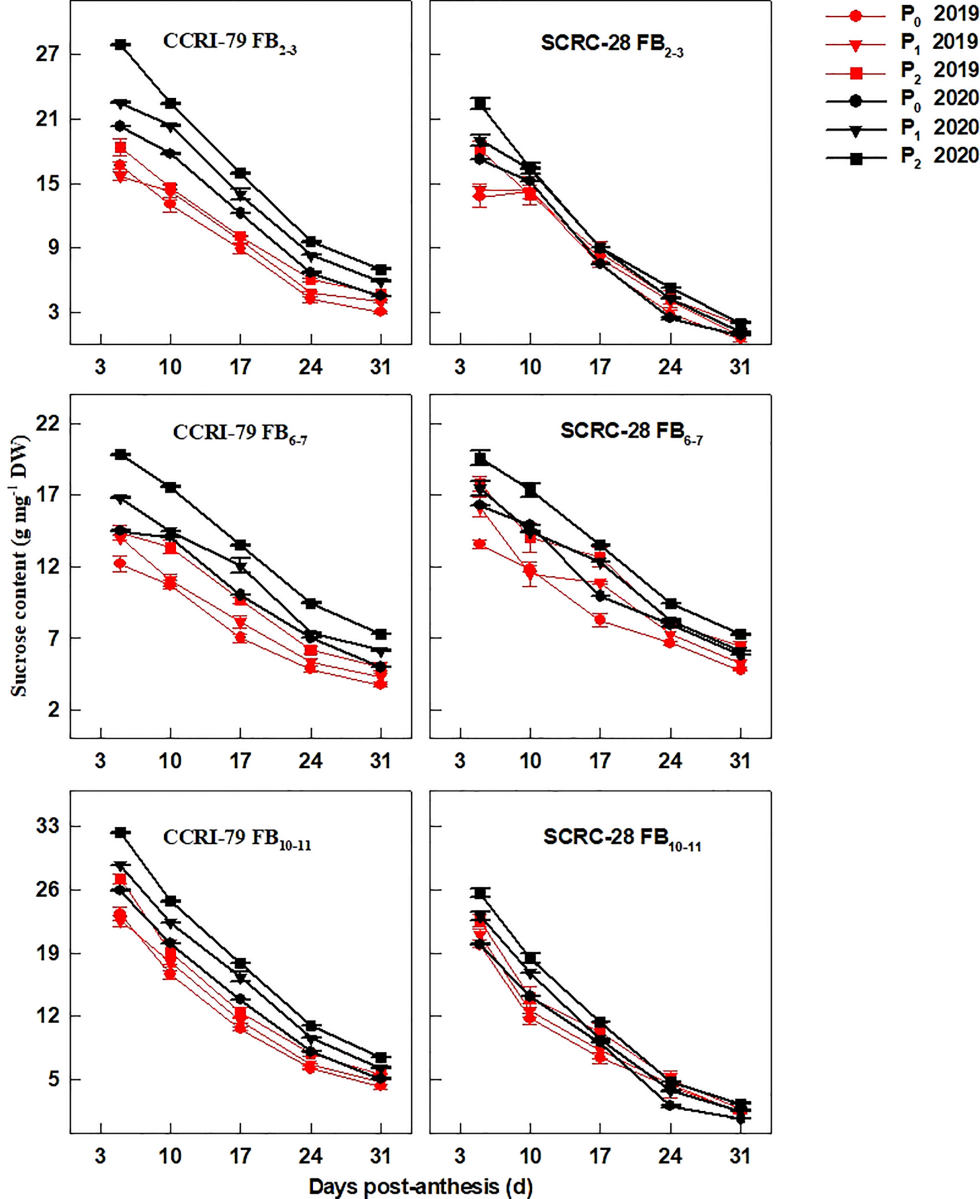
Cotton fiber sucrose content in relation to soil available phosphorus (AP) levels in 2019−2020. FB: fruiting branch. P_0_: 3 ± 0.5 mg kg^−1^. P_1_: 6 ± 0.5 mg kg^−1^. P_2_: 15 ± 0.5 mg kg^−1^. The cotton fibers were collected at 5, 10, 17, 24, and 31 days post-anthesis (DPA) (8:00-9:00 a.m.). Error bars indicate SE (n = 3).

Interestingly, the fiber sucrose content had a similar trend with the soluble sugar content during the fiber elongation progress (**Figure 4**). During the course of 24 months, under P_1_ and P_0_, the sucrose content at the three FBPs was reduced by 10.1−14.2% and 19.0−20.4% in CCRI-79 and by 10.1−13.2% and 19.3−22.2% in SCRC-28, respectively. Moreover, the fibers of FB_10–11_ registered lower decline rates of the sucrose content than FB_2–3_ and FB_6–7_ for CCRI-79 but higher decline rates for SCRC-28 in 2019 and 2020 (**Figure 4**).

### 3.2 Relationship between P contents and osmotically active solutes contents

**Figure 5:**
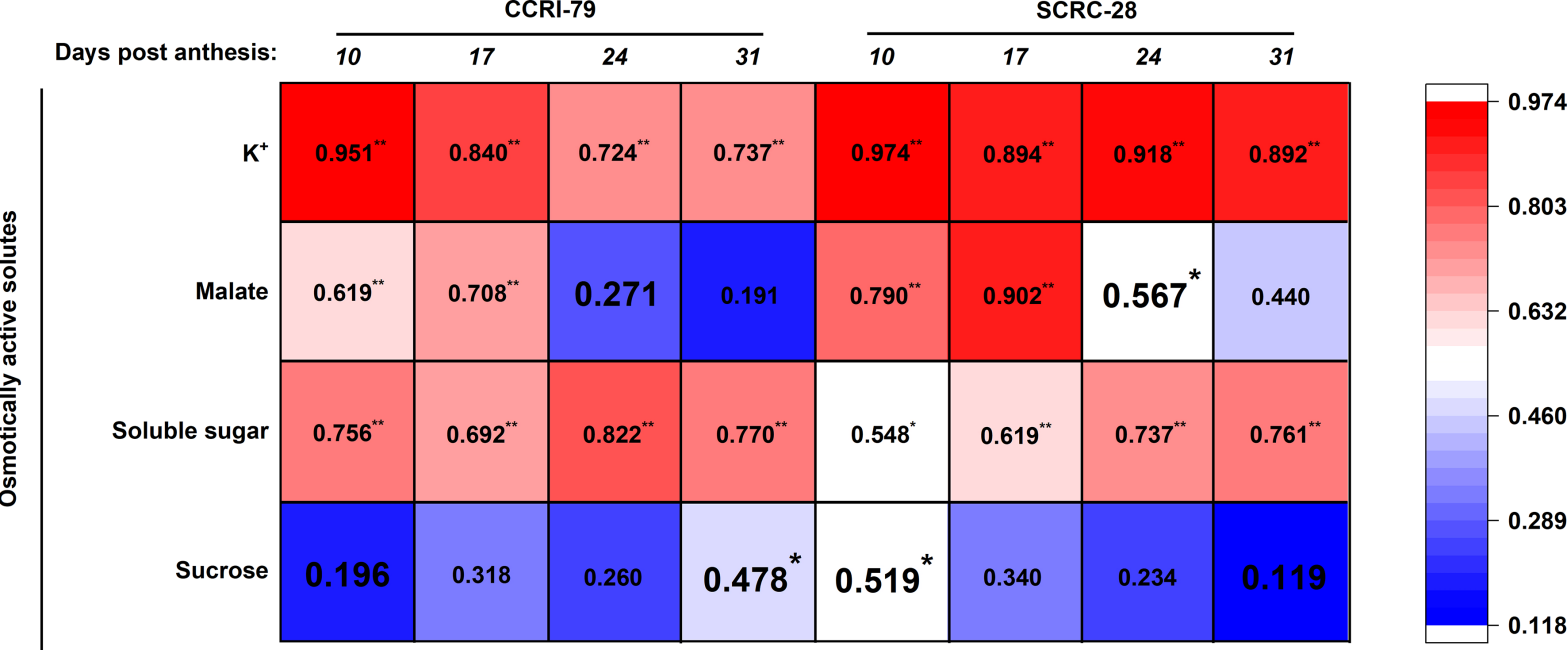
Relationship between osmotically active solute content engaged in fiber elongation and phosphorus (P) contents of the subtending leaves in 2019−2020. The P content data of the subtending leaves were referred from Sun et al. (2022). * and ** represent significant differences at *p*<0.05 and *p*<0.01. n=18, *R*_0.05 _= 0.468, *R*_0.01 _= 0.590.

Positive relations existed between the osmotically active solutes involved in fiber elongation, especially the K^+^ and soluble sugar contents, and P content of subtending leaves of both cultivars at 10−31 DPA (*p*<0.05) (**Figure 5**). Significantly positive correlations were also observed between these contents from 10 to 24 DPA (*p*<0.05) (except for malate content of CCRI-79 at 24 DPA). The correlation between the P content of the leaves and the sucrose content of the fibers were all significantly positive (*p*<0.05) at 10 DPA for SCRC-28 and 31 DPA for CCRI-79 (**Figure 5**).

### 3.3 Activities of enzymes associated with fiber elongation

**Figure 6:**
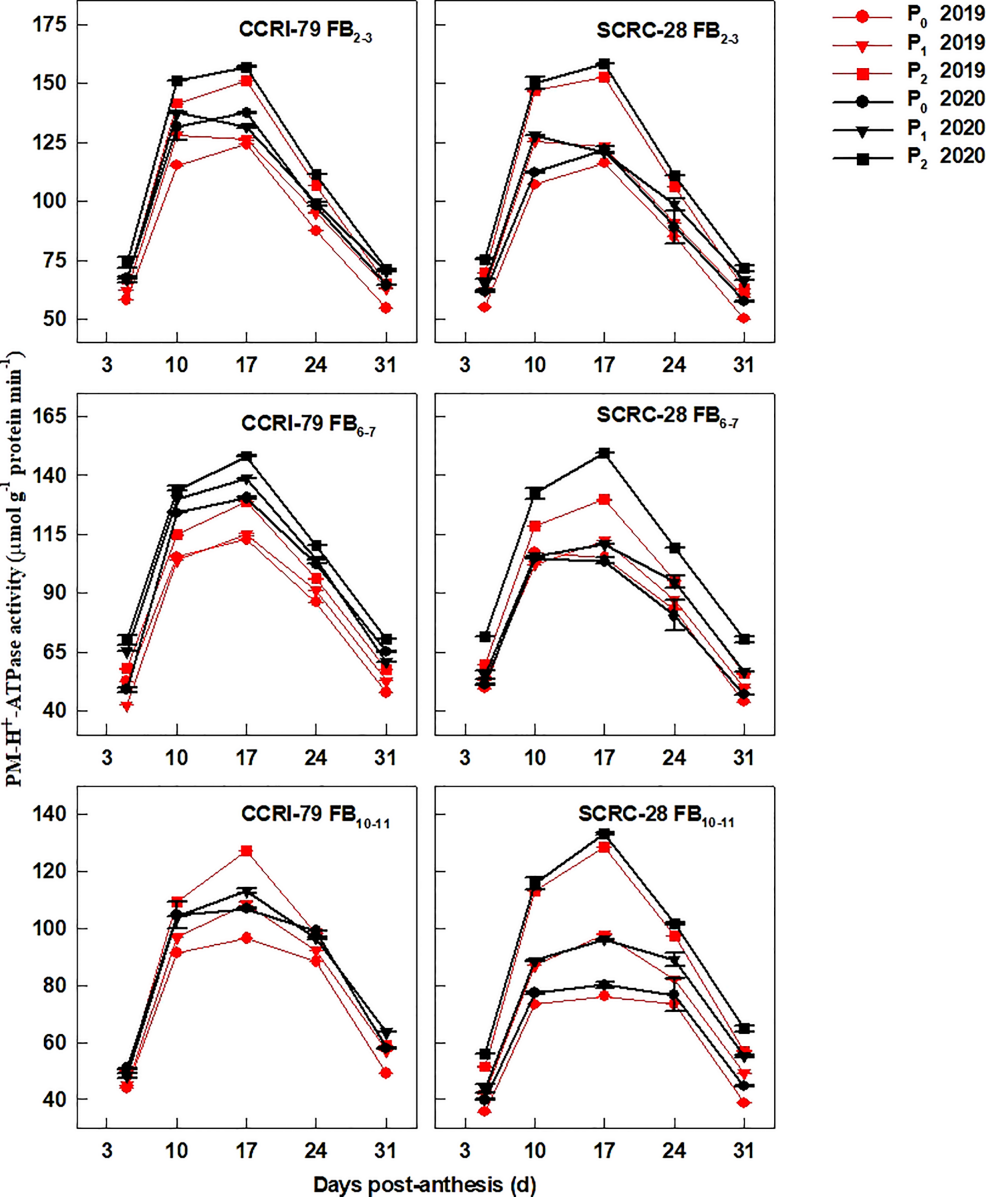
The H^+^-ATPase activities of the cotton fiber plasma membrane (PM) (PM-H^+^-ATPase) in relation to soil available phosphorus (AP) levels in 2019−2020. FB, fruiting branch. P_0_: 3 ± 0.5 mg kg^−1^. P_1_: 6 ± 0.5 mg kg^−1^. P_2_: 15 ± 0.5 mg kg^−1^. The cotton fibers were collected at 5, 10, 17, 24, and 31 days post-anthesis (DPA) (8:00-9:00 a.m.). Error bars indicate SE (n = 3).

**Figure 7:**
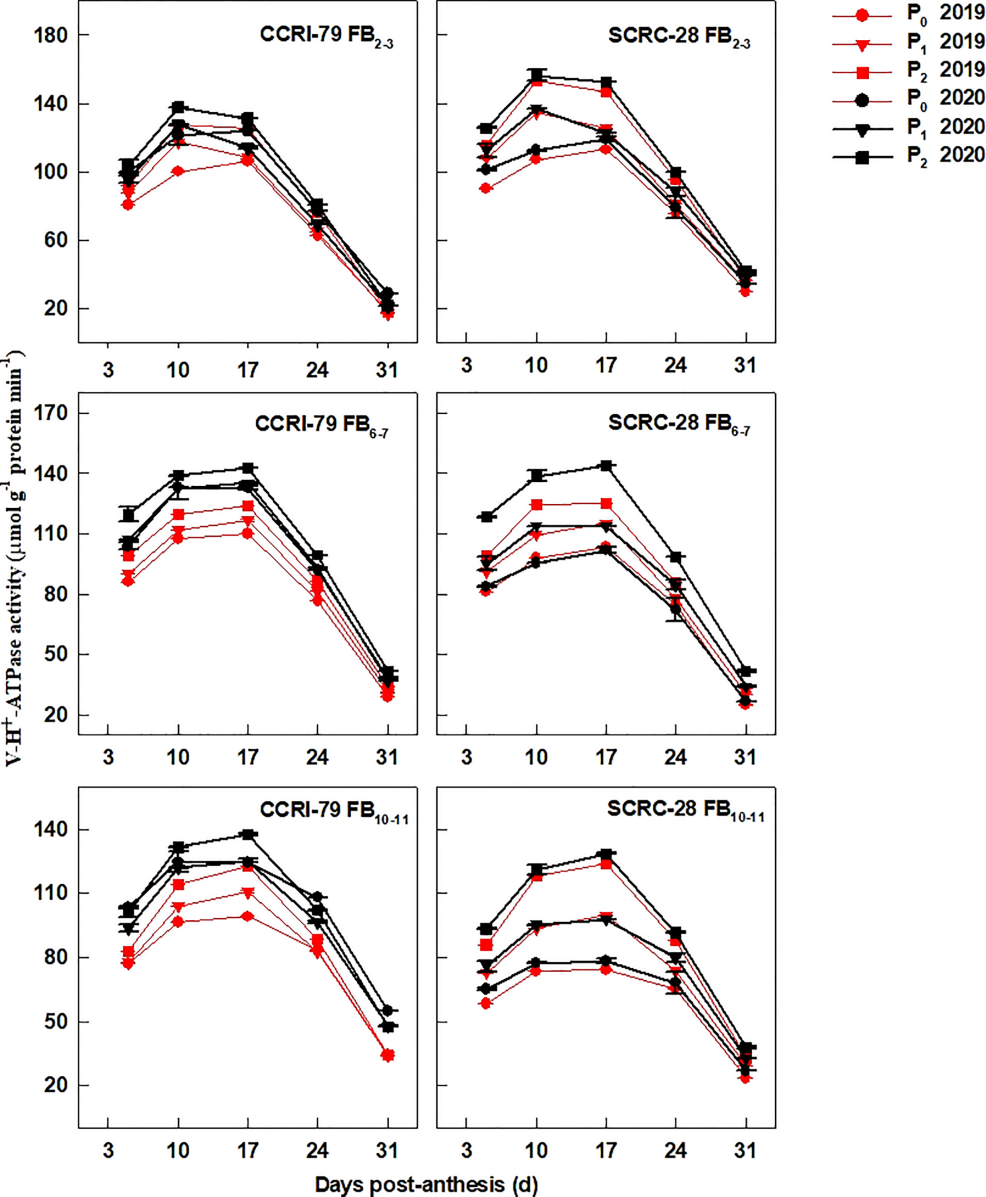
The H^+^-ATPase activities of the cotton fibers vacuole membrane (V-H^+^-ATPase) in relation to soil available phosphorus (AP) levels in 2019-2020. FB, fruiting branch. P_0_: 3 ± 0.5 mg kg^−1^. P_1_: 6 ± 0.5 mg kg^−1^. P_2_: 15 ± 0.5 mg kg^−1^. The cotton fibers were collected at 5, 10, 17, 24, and 31 days post-anthesis (DPA) (8:00-9:00 a.m.). Error bars indicate SE (n = 3).

**Figure 8:**
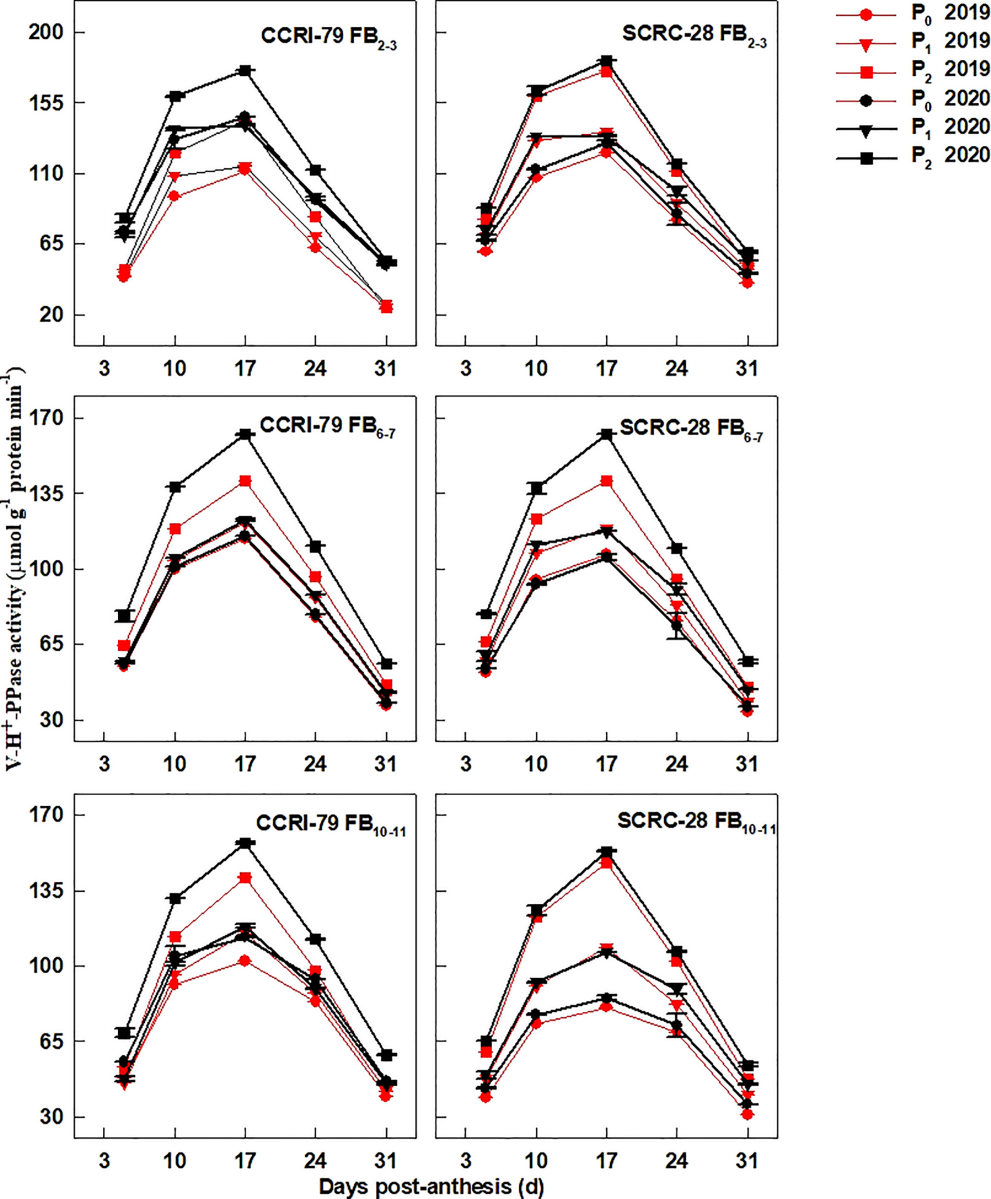
The H^+^-translocating inorganic pyrophosphatase (H^+^-PPase) activities of the cotton fibers vacuole membrane (V-H^+^-PPase) in relation to soil available phosphorus (AP) levels in 2019−2020. FB, fruiting branch. P_0_: 3 ± 0.5 mg kg^−1^. P_1_: 6 ± 0.5 mg kg^−1^. P_2_: 15 ± 0.5 mg kg^−1^. The cotton fibers were collected at 5, 10, 17, 24, and 31 days post-anthesis (DPA) (8:00-9:00 a.m.). Error bars indicate SE (n = 3).

The PM-H^+^-ATPase (**Figure 6**), V-H^+^-ATPase (**Figure 7**), and V-H^+^-PPase (**Figure 8**) activities of fiber presented similar trends reaching its maximum at 17 DPA and declining with increasing soil AP content. Under P_1_ and P_0_, the PM-H^+^-ATPase activity increased by 8.4−10.5% and 11.3−14.4% for CCRI-79 and by 14.9−20.5% and 21.8−33.0% for SCRC-28, respectively at all the FBPs in the two years compared to P_2_ (**Figure 6**). As compared to SCRC-28, CCRI-79 exhibited 8.5 and 15.8% higher activity of fiber PM-H^+^-ATPase under P_1_ and P_0_, but 0.6% lower under P_2_ (**Figure 6**). The V-H^+^-ATPase activities reduced by 7.0−9.8% and 5.9−10.4% for CCRI-79, and by 12.4−18.6% and 23.4−33.8% for SCRC-28 under P_1_ and P_0_, respectively, in contrast with P_2_ treatments throughout the FBPs and growing seasons (**Figure 7**). Nevertheless, under P_1_ and P_0_, the V-H^+^-PPase activities decreased by 14.1−19.4% and 17.2−20.5% in CCRI-79, and by 18.3−23.7% and 28.5−38.4% in SCRC-28, respectively, in contrast with P_2_ treatments throughout the FBPs and the two years (**Figure 8**).

**Figure 9:**
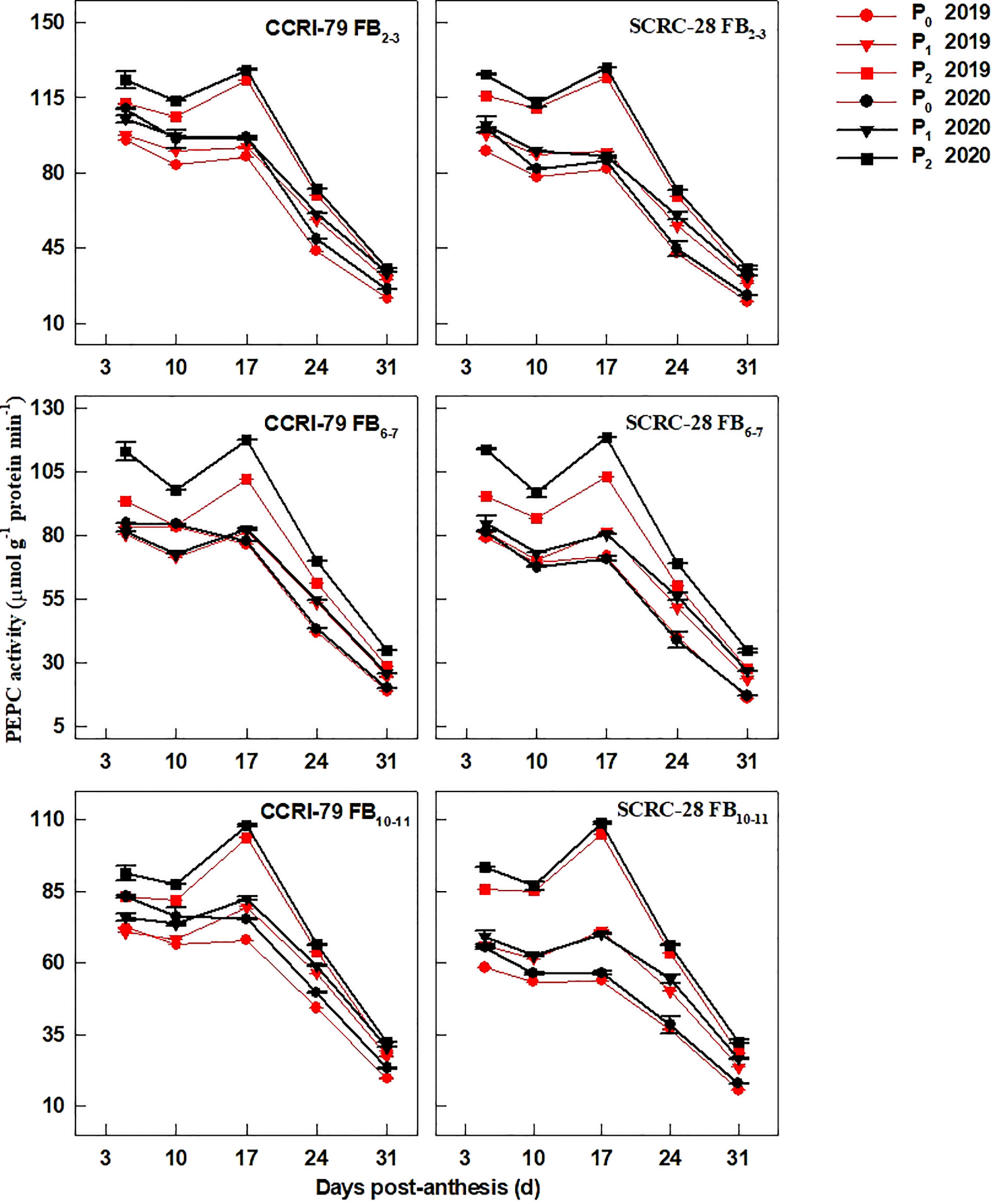
The phosphoenolpyruvate carboxylase (PEPC) activities of the cotton fibers in relation to soil available phosphorus (AP) levels in 2019 and 2020. FB, fruiting branch. P_0_: 3 ± 0.5 mg kg^−1^. P_1_: 6 ± 0.5 mg kg^−1^. P_2_: 15 ± 0.5 mg kg^−1^. The cotton fibers were collected at 5, 10, 17, 24, and 31 days post-anthesis (DPA) (8:00-9:00 a.m.). Error bars indicate SE (n = 3).

The fiber PEPC activity presented a single-peaked curve during fiber elongation and reached its climax at 17 DPA (**Figure 9**). Compared to P_2_, the PEPC activity for CCRI-79 reduced by 16.6−21.7% and 22.6−23.4% in P_1_ and P_0_ at the three FBPs in 2019−2020. Similarly, in P_1_ and P_0_, the PEPC activity reduced by 20.9−26.5% and 30.0−40.2% for SCRC-28. Low soil AP levels (P_1_ and P_0_) reduced the PEPC activities of fibers in the FB_10–11_ compared to FB_2–3_ and FB_6–7_ in SCRC-28 but had less effect on the three FBPs of CCRI-79. Obviously, the variations in the four enzymes involved in fiber elongation of SCRC-28 were greater than those of CCRI-79 under low-P-stress.

### 3.4 Interrelationship between P contents and key enzymes activities

**Figure 10:**
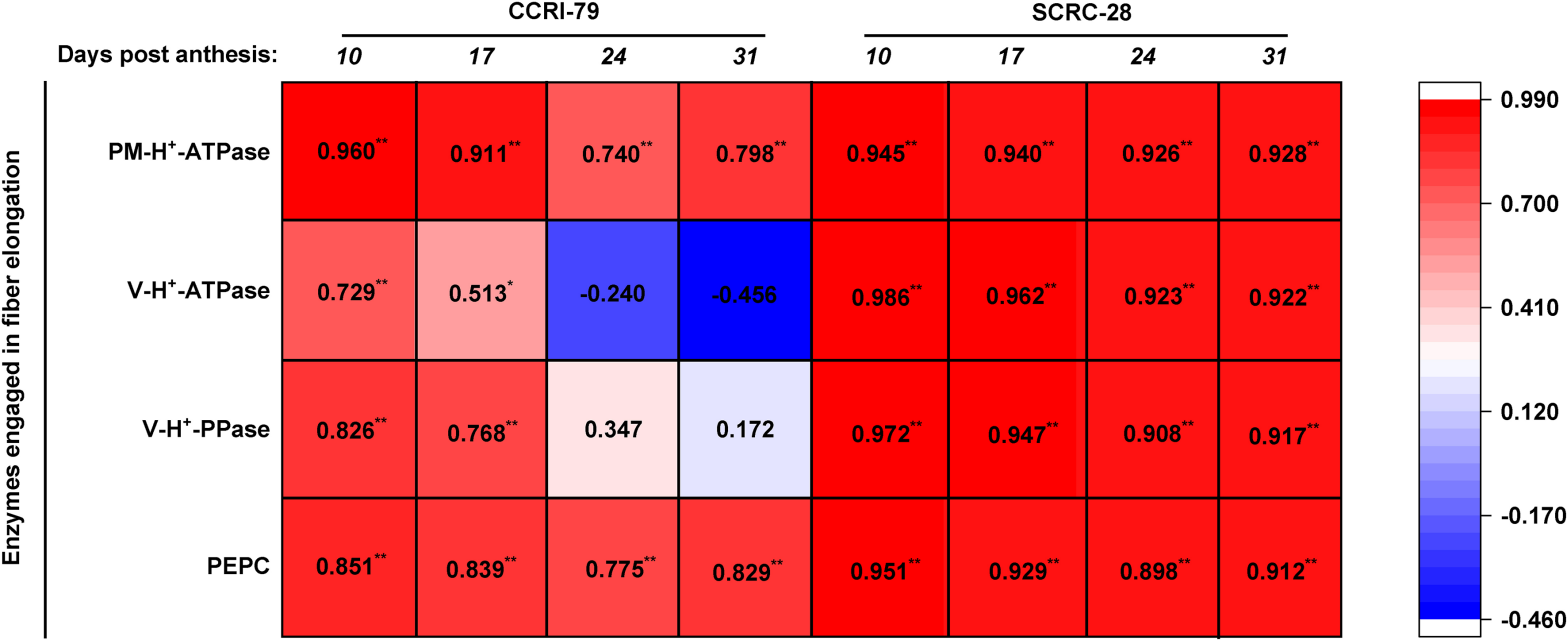
Interrelationship between the activities of the enzymes engaged in fiber elongation and phosphorus (P) contents of the subtending leaves in 2019−2020. The P content data of the subtending leaves were referenced from Sun et al. (2022). * and ** show significant differences at *p*<0.05 and *p*<0.01. n=18, *R*_0.05 _= 0.468, *R*_0.01 _= 0.590.

Among the two cultivars, the enzymes involved in fiber elongation and P content were positively correlated, peculiarly PM-H^+^-ATPase and PEPC activities (*p*<0.01), during fiber elongation (**Figure 10**). The positive correlations between P contents, and the V-H^+^-ATPase activities and V-H^+^-PPase were very significant (*p*<0.01) at 10−31 DPA in SCRC-28, and significant (*p*<0.05) at 10−17 DPA in CCRI-79 (**Figure 10**).

### 3.5 Dynamic changes in the fiber length on the different fruiting branches

**Figure 11:**
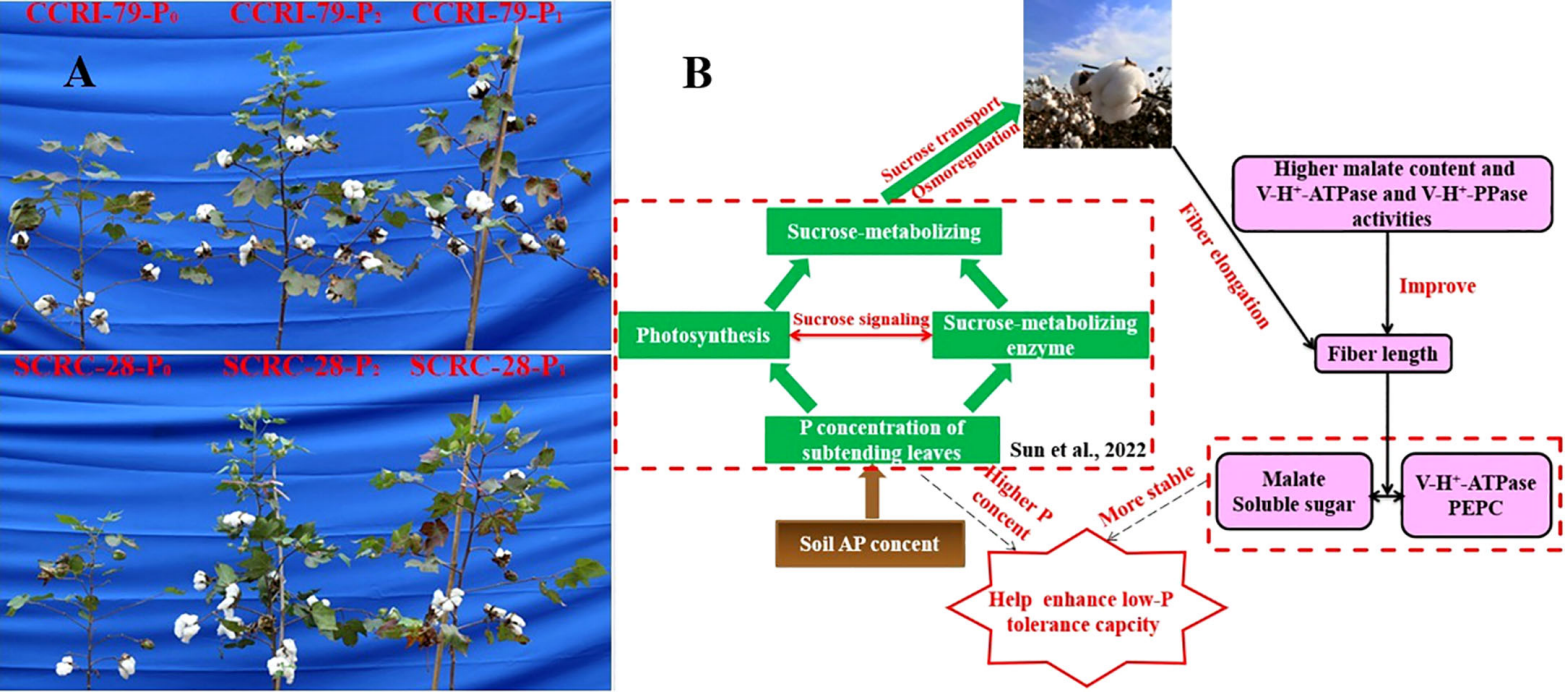
The growth phenotype of CCRI-79 and SCRC-28 under three soil available phosphorus (AP) levels **(A)** and the schematic diagram shows the mechanism of how soil AP deficiency affects fiber elongation **(B)**.

The lack of AP in soil seriously hindered the growth and development of cotton plants (**Figure 11A**), thereby affecting fiber length (**Table 2**). The fiber length changes formed an “s-shape” curve during the development of cotton bolls, which could be fitted using the logistic equation (Yang et al., 2016) as follows:

**Table 2:** Elongation eigenvalues influenced by soil phosphorus availability (AP) in 2019−2020.

			2019					2020				
Cultivar	FBP	AP Treatment	R^2^	V_Lmax_	T_L_	L_max_	L_obs_	R^2^	V_Lmax_	T_L_	L_max_	L_obs_
				(mm d^-1^)	(d)	(mm)	(mm)		(mm d^-1^)	(d)	(mm)	(mm)
CCRI-79	FB_2-3_	P_0_	1.000	2.4	7.6	28.0	27.8b	0.999	2.2	8.4	27.6	27.6b
		P_1_	1.000	2.5	7.5	28.6	28.7ab	0.999	2.3	8.2	28.6	28.3ab
		P_2_	0.999	2.7	7.4	30.2	29.6a	0.999	2.4	7.9	29.4	29.5a
		CV (%)	0.1	6.0	1.3	3.9	3.1	0.0	4.3	3.1	3.2	3.4
	FB_6-7_	P_0_	1.000	2.5	7.6	28.3	28.3b	0.999	2.2	8.4	28.6	28.6b
		P_1_	0.999	2.6	7.2	28.9	29.3a	0.999	2.4	8.2	29.3	29.3ab
		P_2_	0.999	2.7	7.3	29.9	29.6a	0.999	2.5	7.9	30.1	30.3a
		CV (%)	0.1	3.8	2.8	2.8	2.4	0.0	6.5	3.1	2.6	2.9
	FB_10-11_	P_0_	1.000	2.5	7.6	28.3	28.1b	0.999	2.3	8.4	28.9	28.9b
		P_1_	1.000	2.5	7.5	28.0	28.8a	0.999	2.4	8.2	29.9	29.6ab
		P_2_	0.999	2.6	7.4	29.5	29.3a	0.999	2.5	7.9	30.0	30.1a
		CV (%)	0.1	2.3	1.3	2.8	2.0	0.0	4.2	3.1	2.1	2.1
SCRC-28	FB_2-3_	P_0_	0.998	2.0	8.3	25.2	25.4b	0.999	2.1	8.4	26.9	26.7b
		P_1_	0.994	2.4	7.9	28.1	28.2a	1.000	2.3	8.3	28.2	28.1a
		P_2_	0.995	2.5	7.8	29.4	29.1a	0.999	2.4	7.9	29.2	28.9a
		CV (%)	0.2	11.5	3.3	7.8	6.9	0.1	6.7	3.2	4.1	4.1
		P_0_	0.998	2.1	8.3	26.7	26.9c	0.999	2.2	8.4	28.1	27.8 b
		P_1_	0.994	2.3	7.9	27.9	28.0b	1.000	2.3	8.4	29.5	29.3ab
		P_2_	0.995	2.5	7.7	29.2	28.9a	0.999	2.5	7.9	30.0	29.7 a
		CV (%)	0.2	8.7	3.8	4.5	3.6	0.1	6.5	3.5	3.4	3.5
	FB_10-11_	P_0_	0.998	2.1	8.3	26.6	26.8b	0.999	2.2	8.4	27.7	27.4b
		P_1_	0.994	2.3	7.9	27.2	27.3b	1.000	2.3	8.3	28.9	28.8a
		P_2_	0.996	2.4	7.8	28.8	28.6a	0.999	2.5	7.9	29.5	29.2a
		CV (%)	0.2	6.7	3.3	4.1	3.4	0.1	6.5	3.2	3.2	3.3
Significance												
Cultivar (C)							**					**
FBP							NS					**
AP							**					**
C×FBP							NS					NS
C×AP							*					NS
FBP×AP							*					NS
C×FBP×AP							NS					NS

FBP, fruiting branch position; CV, coefficient of variation; V_Lmax_, the maximum velocity of ﬁber elongation; T_L_, ﬁber rapid elongation duration; L_max_, theoretical maximum ﬁber length; L_obs_, observed ﬁnal ﬁber length. Different letters within a column represent significant differences at p=0.05. * and ** represent significant differences at p<0.05 and p<0.01. NS represents nonsignificance at p = 0.05.


(1) $$L=\frac{L_{\max}}{1+a\times e^{b\times DAP}}$$

L means the fiber length (mm), L_max_ means the theoretical maximum fiber length, and a and b mean parameters.

The maximum velocity of fiber elongation (V_Lmax_), the beginning time of rapid elongation (T_B_), the finishing time of rapid elongation (T_F_), and the rapid elongation duration (T_L_=T_F_-T_B_) of cotton fibers were calculated using formulas (2), (3), and (4), respectively.


(2) $${\text{V}}_{\text{Lmax}}=\frac{\text{-b}\times {\text{L}}_{\text{max}}}{4}$$

(3) $$T_{B}=\frac{1}{b}\times \ln\frac{2+\sqrt{3}}{a}$$

(4) $$T_{F}=\frac{1}{b}\times \ln\frac{2-\sqrt{3}}{a}$$

**Figure 12:**
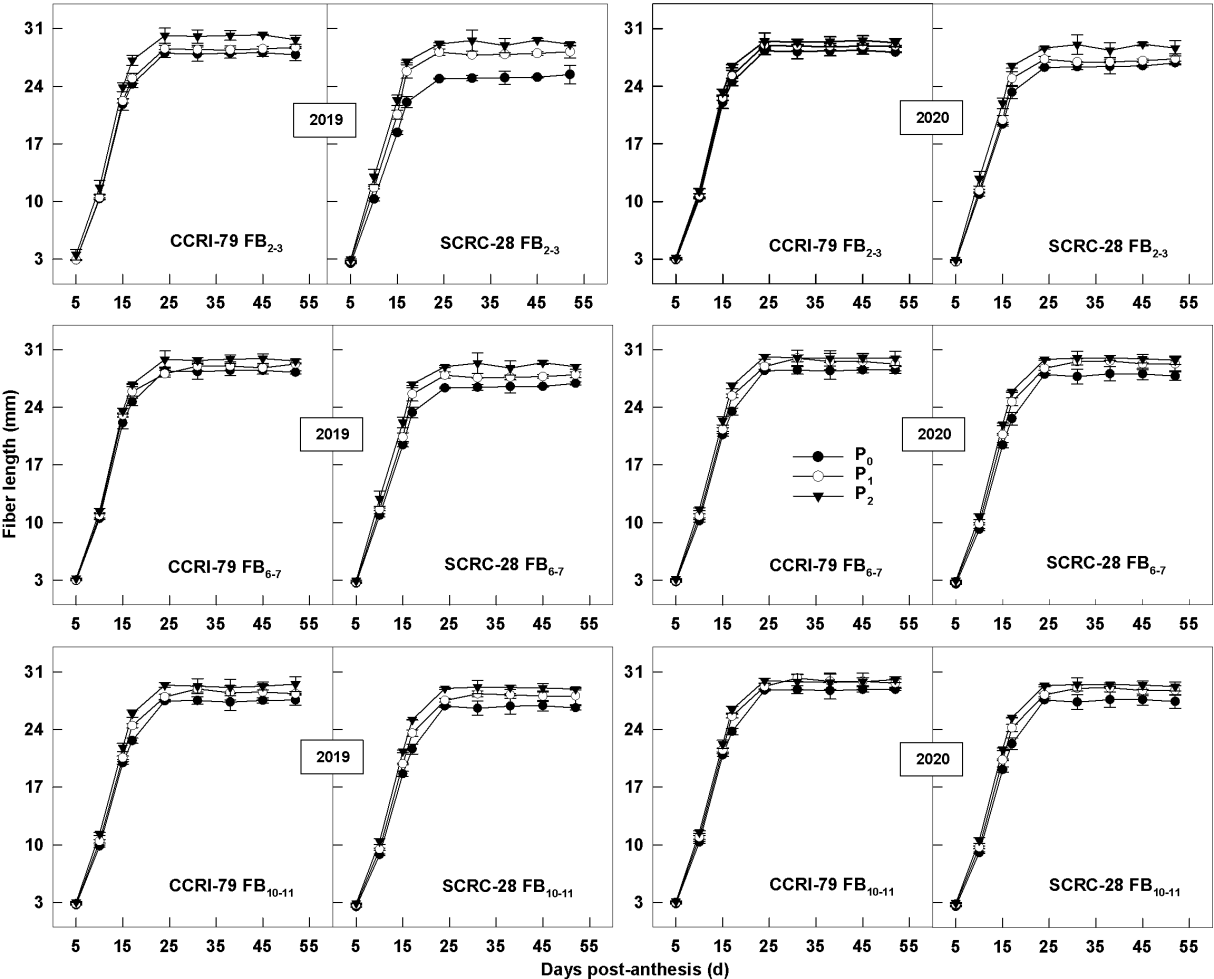
Dynamic changes of cotton fiber lengths in relation to soil available phosphorus (AP) levels in 2019−2020. FB, fruiting branch. P_0_: 3 ± 0.5 mg kg^−1^. P_1_: 6 ± 0.5 mg kg^−1^. P_2_: 15 ± 0.5 mg kg^−1^. The cotton fibers were collected at 5, 10, 15, 17, 24, 31, 38, 45 and 52 days post-anthesis (DPA) (8:00-9:00 a.m.). Error bars indicate SE (n = 3).

The rapid elongation stage of the fibers started from 10 to 24 DPA, after which the fiber length tended to stabilize (**Figure 12**). Low soil AP levels (P_0_ and P_1_) decreased the V_Lmax_, but increased the T_L_, ultimately reducing the fiber length. The fiber length variation was consistent for CCRI-79 and SCRC-28 over the two years (**Table 2**). V_Lmax_ had higher sensitivity to soil AP deficiency than T_L_. Compared to P_2_, the fiber length reduced by 2.2–3.6% and 3.9–6.3% in P_1_ and P_0_ for CCRI-79, mainly because V_Lmax_ declined by 3.8–5.9% and 5.9–9.8% in 2019 and 2020 across the FBPs. For SCRC-28, the fiber length reduced by 2.2–3.8% and 6.7–10.2% at P_1_ and P_0_ in contrast with P_2_ due to the fact that V_Lmax_ reduced by 4.1–6.1% and 12.2–16.3% for both years at the three FBPs. In P_1_ and P_0_, CCRI-79 had a fiber length that was 2.3 percent and 5.2 percent longer than SCRC-28. However, CCRI-79 fiber length was 2.1% longer than SCRC-28 over the two-year under P_2_ treatment. Moreover, there were longer fibers for CCRI-79 than those for SCRS-28 in soil AP deficiency.

The CVs of fiber length were lower in FB_10–11_ than in FB_2–3_ and FB_6–7_ for both cultivars, and the values were even smaller for CCRI-79. Cultivar (C) (*p*<0.01) and AP (*p*<0.01) had the greatest impacts on fiber length (**Table 2**).

### 3.6 Correlation between physiological parameters and fiber length

**Figure 13:**
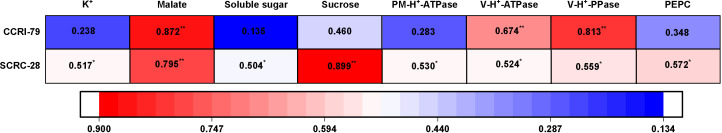
Correlation between the contents of the osmotically active solutes and the activities of enzymes engaged in fiber elongation and fiber length in 2019−2020. * and **represent significant differences at *p*<0.05 and *p*<0.01. n=18, *R*_0.05 = _0.468, *R*_0.01 = _0.590.

We determined the cotton fiber lengths and their relationship with the contents of the osmotically active solutes and the activities of the enzymes engaged in fiber elongation. The results showed that fiber lengths had significant positive correlations (*p*<0.05) with the contents of all osmotically active solutes (K^+^, malate, soluble sugar, and sucrose) in SCRC-28 but correlated only with malate contents in CCRI-79 (*p*<0.01) (**Figure 13**). Moreover, fiber lengths were significantly positively correlated (*p*<0.01) with the V-H^+^-ATPase and V-H^+^-PPase activities in CCRI-79 (**Figure 13**). There was a remarkable positive correlation between fiber length and related enzyme activities in SCRC-28 as well (*p*<0.05) (**Figure 13**).

## 4 Discussion

P application on cotton fiber length may have varying effects (Mukundan et al., 1990; Tewolde and Fernandez, 2003; Li et al., 2020), possibly due to the influence of soil AP content. We analyzed soil nutrient data and found that soil AP content is the main factor affecting fiber development and length; therefore, we used low soil AP levels to induce P deficiency stress in cotton plants. Our previous study showed that low soil AP affects sucrose transport and synthesis in the subtending leaves, thereby reducing cotton boll biomass and lint yield (Sun et al., 2022). Therefore, the current research explored the soil AP content’s effect on cotton fiber elongation and length, which could more precisely illustrate the P status of the cotton bolls and the subtending leaves.

P fertilizer increased the K^+^ content of cotton plants (including roots, stems, and leaves) (Luo et al., 2017) and the root malate content of alfalfa seedlings (Wang et al., 2021). In the experiment, the K^+^ contents of cotton fibers decreased by 2.9 and 6.5% under P_1_ and P_0_ conditions, respectively, compared to P_2_ (**Figure 1**). It indicated that soil AP deficiency hindered K absorption, thereby reducing the K^+^ content of fibers, as reported in a previous study (Luo et al., 2017). However, some studies have shown that excessive P application can reduce the K^+^ content of lettuce (Chen et al., 2015) and grass (Sabreen et al., 2022) due to the diluting effects caused by increased plant yield. The malate contents of the cotton fibers reduced by 19.7 and 30.6% in P_1_ and P_0_, compared to P_2_ (**Figure 2**), indicating that low soil AP limited the malate synthesis, consistent with previous reports (Fernandez Del-Saz et al., 2017). Compared with 2019, the changes in soluble sugar content were greater under three soil AP levels in 2020. It may be related to precipitation (**Table 1**), and drought can exacerbate the impact of low-P-stress (Singh et al., 2006a; Singh et al., 2006b). Low soil AP limited the transportation of photosynthetic products to cotton bolls (lower sucrose transformation rate) (Sun et al., 2022), further reducing the soluble sugar and sucrose contents of the fibers (**Figures 3**, **4**). K^+^, malate, soluble sugar, and sucrose are important osmotically active solutes facilitating fiber elongation (Ruan et al., 1997; Ruan et al., 2001). The K^+^, malate, soluble sugar, and sucrose contents of the cotton fibers were positively correlated with the P content of the subtending leaves at 10−31 DPA (**Figure 5**). This indicates that low soil AP reduces the contents of osmotically active solutes in the fibers by affecting P content and sucrose metabolism in the subtending leaves, further influencing fiber vacuoles which facilitate fiber elongation through osmoregulation (Yang et al., 2016).

Low-P-stress increases root PM-H^+^-ATPase activities (Shen et al., 2006) and promotes organic acid secretion (Yan et al., 2002) to enhance P absorption capacity in crops. However, in our experiment, the activities of fiber PM-H^+^-ATPase declined by 13.4 and 19.5% under P_1_ and P_0_ (soil AP deficiency), respectively, compared to P_2_ (**Figure 6**). A significant positive correlation (*p*<0.01) was found between the fiber PM-H^+^-ATPase activity and the P content of the subtending leaves during the period of 10–31 DPA (**Figure 10**), similar to the report by Salinas et al. (2013). This indicated that soil AP content has an important regulatory effect on PM-H^+^-ATPase activity. It also points out that there are differences in the response of PM-H^+^-ATPase activity to low-P-stress among crop species, and PM-H^+^-ATPase is mainly responsible for generating transmembrane electrochemical gradient to drive the transportation of many substances, which may be related to higher P levels (Chang et al., 2009). Additionally, the responses of fiber V-H^+^-ATPase, V-H^+^-PPase, and PEPC activities (**Figures 7**–**9**) to soil AP deficiency were similar to that of PM-H^+^-ATPase activity (**Figure 6**). The P content of the subtending leaves affects the activity of enzymes related to cotton fiber elongation in various ways. P levels can alter the affinities between enzymes and substrates (Xu et al., 2008), and low-P-stress can affect enzyme activities through the sucrose signaling pathways (Wang and Ruan, 2013; Ruan, 2014). Moreover, the impact of low-P-stress on the “source (subtending leaf)” may lead to insufficient sucrose supply in the “sink (cotton fiber)” (Sun et al., 2022). This may trigger sucrose signaling leading to downregulation of the upstream genes coding for the related enzymes involved in fiber elongation (Wang and Ruan, 2013; Ruan, 2014), thus limiting the catalytic abilities of the enzymes at the genetic level. In our study, we verified that low soil AP level play the osmoregulation role in fiber elongation through the P content of subtending leaves (**Figure 11B**) (Yang et al., 2016).

After analyzing the two-year V_Lmax_ and T_L_ data for the CCRI-79 and SCRC-28, we found that the CVs (6.0%) of the V_Lmax_ were greater than twice that of T_L_ (2.8%) (**Table 2**), indicating that soil AP deficiency mainly reduces fiber length (**Figure 12**) by decreasing the V_Lmax_ (Yang et al., 2016). Soil AP deficiency limited the transportation of the osmotically active solutes from subtending leaves to fibers, reducing the activity of related enzymes involved in fiber elongation, thus inhibiting fiber elongation and ultimately reducing fiber length. Moreover, low-P-stress, especially the P_0_ treatment, highly impacted the fiber length of FB_2–3_ more than FB_10–11_. This was because the CVs of the osmotically active solutes (K^+^, malate, soluble sugar, and sucrose) and related enzymes engaged in fiber elongation (PM-H^+^-ATPase, V-H^+^-ATPase, V-H^+^-PPase, and PEPC) were lower at FB_10–11_ than at FB_2–3_.

The agronomic and yield traits of different cotton varieties have different sensitivities to low P (Iqbal et al., 2020; Li et al., 2020); however, it is unclear whether cotton fiber length also has varying sensitivities to low P. In our research, soil AP deficiency significantly impacted the contents of the osmotically active solutes (**Figure 5**), activities of related enzymes involved in fiber elongation **Figure 10**), and fiber length (**Figure 12**; **Table 2**) of SCRC-28 and CCRI-79. Furthermore, the responses of these parameters to soil AP deficiency showed that SCRC-28 had higher sensitivity to low-P-stress compared to CCRI-79. Among all the osmotically active solute contents, fiber K^+^ content was the most correlated with leaf P content in CCRI-79 and SCRC-28; whereas, the interrelationship between fiber malate, soluble sugar contents, and fiber length differed between the cultivars (**Figure 5**). The outcomes may elucidate the reason that SCRC-28 was more sensitive to low-P-stress compared to CCRI-79 (**Table 2**), suggesting higher fiber malate content might be critical for fiber length (**Figure 13**) (Ruan et al., 1997; Yang et al., 2016).

In the experiment, the fibers’ V-H^+^-ATPase and V-H^+^-PPase activities were more sensitive to P contents of the subtending leaves of SCRC-28 than CCRI-79 (**Figure 10**). Moreover, the relationship between PM-H^+^-ATPase and PEPC activities and fiber length had differences in the two cultivars (**Figure 13**). The V-H^+^-ATPase activity decreased by 5.9–10.4% for CCRI-79 and 12.4–33.8% for SCRC-28 in P_1_ and P_0_ at all the FBPs. Moreover, the V-H^+^-ATPase and V-H^+^-PPase activities of the fibers had significant positive correlations with the fiber length (*p*<0.05) in both cultivars. Compared with P_2_, in P_1_ and P_0_ the PEPC activity in fibers reduced by 16.6–23.4% for CCRI-79 and 20.9–40.2% for SCRC-28, respectively, over the course of the three FBPs and two years. And the above results revealed that the SCRC-28’s V-H^+^-ATPase and PEPC activities had higher sensitivity to low soil AP levels, explaining the sensitivity of the fiber length of the low-P sensitive cultivars to soil AP deficiency.

## 5 Conclusions

1. Low soil AP levels (P_0_ and P_1_) inhibited the fiber cell elongation leading to reduced V_Lmax_ and fiber length, mainly due to lower malate content and V-H^+^-ATPase and V-H^+^-PPase activities.
2. Reduced osmotically active solute contents (K^+^, malate, soluble sugar, and sucrose) and the activities of the related enzymes (PM-H^+^-ATPase, V-H^+^-ATPase, V-H^+^-PPase, and PEPC) involved in fiber elongation was lower at FB_10–11_ than FB_2–3_ under soil AP deficiency (especially P_0_), suggesting that the longer fiber lengths on the upper FBPs (FB_10–11_) could adapt to low soil AP compared to lower FBPs (FB_2–3_).
3. Compared to CCRI-79, the fiber malate and soluble sugar contents and V-H^+^-ATPase and PEPC activities of SCRC-28 were more affected strongly by subtending leaves’ P content, which may elucidate that SCRC-28 shows greater sensitivity to soil AP deficiency.
4. Analyzing the impact of soil low AP on fiber osmoregulation is beneficial for developing low-P-tolerant cotton cultivars. Furthermore, it is necessary to investigate the effects of low-P on fiber gene expression and protein synthesis.

## Data availability statement

The original contributions presented in the study are included in the article/supplementary material. Further inquiries can be directed to the corresponding authors.

## Author contributions

MS: Conceptualization, Funding acquisition, Investigation, Methodology, Writing – original draft. CZ: Conceptualization, Investigation, Writing – original draft. WF: Formal Analysis, Writing – original draft. JS: Formal Analysis, Writing – original draft. CP: Writing – review & editing. PL: Conceptualization, Writing – review & editing. HD: Conceptualization, Writing – review & editing, Funding acquisition.
